# *N*-[2-({[1-(4-Chloro­phen­yl)-1*H*-pyrazol-3-yl]­oxy}meth­yl)phen­yl]-*N*-meth­oxy­hydrazinecarboxamide

**DOI:** 10.1107/S1600536812038214

**Published:** 2012-09-12

**Authors:** Rajni Kant, Vivek K. Gupta, Kamini Kapoor, Chetan S. Shripanavar, Kaushik Banerjee

**Affiliations:** a X-ray Crystallography Laboratory, Post-Graduate Department of Physics & Electronics, University of Jammu, Jammu Tawi 180 006, India; b National Research Centre for Grapes, Pune 412 307, India

## Abstract

In the title compound, C_18_H_18_ClN_5_O_3_, the hydrazinecarboxamide N—N—C(O)—N unit is nearly planar [maximum deviation = 0.074 (2) Å] and is inclined at a dihedral angle of 57.43 (7)° with respect to the plane of the attached benzene ring. The chloro­phenyl group makes dihedral angles of 19.71 (7) and 34.07 (6)° with the pyrazole and benzene rings, respectively. In the crystal, pairs of N—H⋯O hydrogen bonds link the mol­ecules into inversion dimers that are further linked into chains along the *a*-axis direction by N—H⋯N hydrogen bonds. In addition, π–π stacking inter­actions are observed between benzene rings [centroid–centroid distance = 3.680 (1) Å].

## Related literature

For the biological activity of pyraclostrobin (systematic name: methyl *N*-{2-[1-(4-chloro­phen­yl)-1*H*-pyrazol-3-yloxymeth­yl]phen­yl}), see: Esteve-Turrillas *et al.* (2011 ▶); Mercader *et al.* (2008 ▶); Patel *et al.* (2012 ▶). For a related structure, see: Attia *et al.* (2012 ▶).
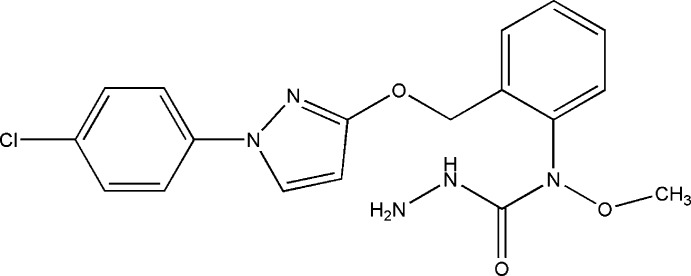


## Experimental

### 

#### Crystal data

C_18_H_18_ClN_5_O_3_
*M* *_r_* = 387.82
Monoclinic, 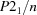

*a* = 7.6830 (4) Å
*b* = 9.1597 (4) Å
*c* = 26.1083 (12) Å
β = 91.683 (4)°
*V* = 1836.55 (15) Å^3^
*Z* = 4
Mo *K*α radiation
μ = 0.24 mm^−1^
*T* = 293 K
0.3 × 0.2 × 0.2 mm

#### Data collection

Oxford Diffraction Xcalibur Sapphire3 diffractometer
Absorption correction: multi-scan (*CrysAlis PRO*; Oxford Diffraction, 2010 ▶) *T*_min_ = 0.832, *T*_max_ = 1.000
27359 measured reflections
3616 independent reflections
2922 reflections with *I* > 2σ(*I*)
*R*_int_ = 0.037

#### Refinement

*R*[*F* ^2^ > 2σ(*F* ^2^)] = 0.048
*wR*(*F* ^2^) = 0.112
*S* = 1.12
3616 reflections
256 parameters
H atoms treated by a mixture of independent and constrained refinement
Δρ_max_ = 0.21 e Å^−3^
Δρ_min_ = −0.23 e Å^−3^

### 

Data collection: *CrysAlis PRO* (Oxford Diffraction, 2010 ▶); cell refinement: *CrysAlis PRO*; data reduction: *CrysAlis PRO*; program(s) used to solve structure: *SHELXS97* (Sheldrick, 2008 ▶); program(s) used to refine structure: *SHELXL97* (Sheldrick, 2008 ▶); molecular graphics: *ORTEP-3* (Farrugia, 1997 ▶); software used to prepare material for publication: *PLATON* (Spek, 2009 ▶).

## Supplementary Material

Crystal structure: contains datablock(s) I, global. DOI: 10.1107/S1600536812038214/gk2517sup1.cif

Structure factors: contains datablock(s) I. DOI: 10.1107/S1600536812038214/gk2517Isup2.hkl

Supplementary material file. DOI: 10.1107/S1600536812038214/gk2517Isup3.cml

Additional supplementary materials:  crystallographic information; 3D view; checkCIF report

## Figures and Tables

**Table 1:** Hydrogen-bond geometry (Å, °)

*D*—H⋯*A*	*D*—H	H⋯*A*	*D*⋯*A*	*D*—H⋯*A*
N5—H51⋯N6^i^	0.88 (2)	2.28 (2)	3.080 (3)	153 (2)
N6—H61⋯O1^ii^	0.86 (2)	2.34 (2)	3.120 (3)	152 (2)
N6—H62⋯N15^ii^	0.87 (3)	2.56 (3)	3.408 (3)	164 (2)

Symmetry codes: (i) 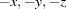; (ii) 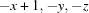.

**Fig. 1.**
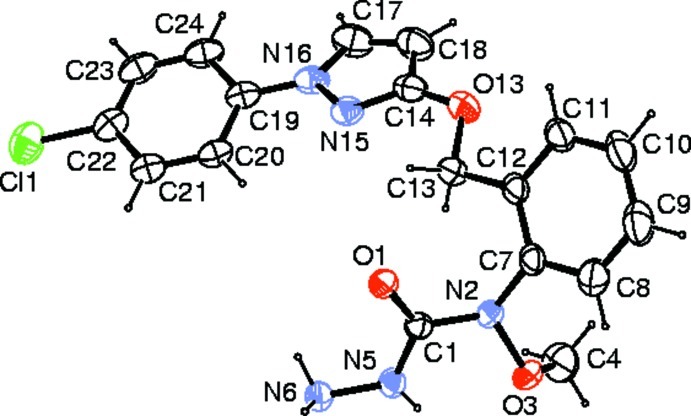
ORTEP view of the molecule with the atom-labeling scheme. The thermal ellipsoids are drawn at the 40% probability level. H atoms are shown as small spheres of arbitrary radii.

**Fig. 2.**
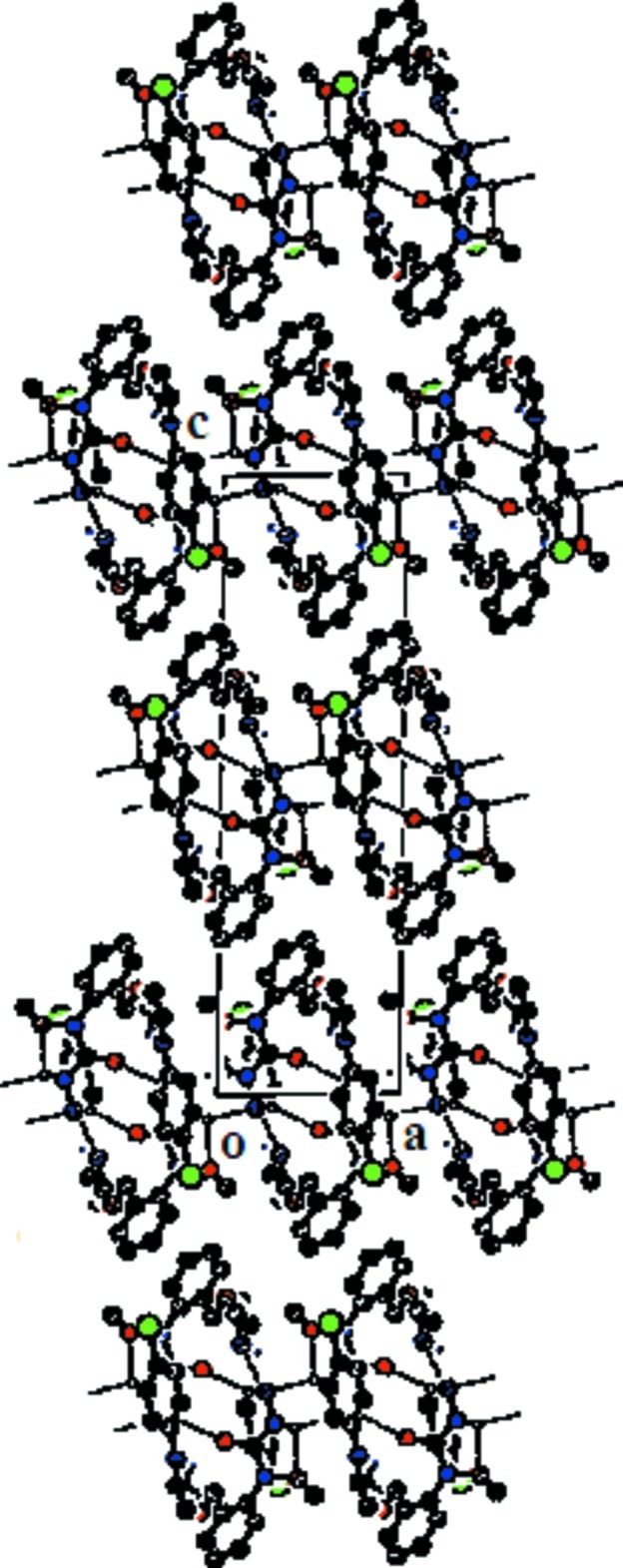
The packing arrangement of molecules viewed down the b axis. The dotted lines show intermolecular N—H···O and N—H···N hydrogen bonds.

## supplementary crystallographic information

### Comment

Pyraclostrobin, which belongs to the latest generation of strobilurin family of fungicides, shows a broad antifungal activity spectrum and higher efficiency and security profiles than previous fungicides (Patel *et al.*, 2012; Esteve-Turrillas *et al.*, 2011; Mercader *et al.*, 2008).

In the title compound all bond lengths and angles are normal and correspond to those observed in the related structure (Attia *et al.*, 2012). The hydrazinecarboxamide moiety (N2,N5,N6/O1/C1) is nearly planar with a maximum deviation of 0.074 (2) Å at atom N6, and is inclined at an angle of 57.43 (7)° with the benzene ring (C7–C12). The dihedral angle between the benzene rings is 34.07 (6)°. Chlorophenyl group makes a dihedral angle of 19.71 (7)° with the pyrazole ring. In the crystal, N6—H61···O1 hydrogen bonds link pairs of molecules to form inversion dimers and dimers are connected *via* N6—H62···N15 and N5—H51···N6 hydrogen bonds to form chains along the *a* axis of the unit cell (Table 1, Fig. 2). The crystal structure is further stabilized by π–π interactions between the benzene ring (C19—C24) of the molecule at (*x*, *y*, *z*) and the benzene ring of an inversion related molecule at (- *x*, 1 - *y*, -*z*)[centroid separation = 3.680 (1) Å, interplanar spacing = 3.396 Å and centroid shift = 1.41 Å].

### Experimental

Pyraclostrobin (0.373 g, 0.001 mol) was dissolved in 5 ml methanol and to it hydrazine hydrate (0.1 g, 0.002 mol) solution was added and refluxed at 70°C for 1 h. The reaction mixture was then cooled and compound was separated out by removal of the solvent under reduced pressure. The product was dissolved in methanol and kept for slow evaporation of solvent to get crystals of the title compound (m.p. 398 K).

### Refinement

The N-bound H atoms were located in a difference Fourier map and freely refined. All other H atoms were positioned geometrically and were treated as riding on their parent C atoms, with C—H distances of 0.93–0.97 Å and with *U*_iso_(H) = 1.2*U*_eq_(C) or 1.5*U*_eq_(methyl C).

### Crystal data

**Table:**

C_18_H_18_ClN_5_O_3_	*F*(000) = 808
*M**_r_* = 387.82	*D*_x_ = 1.403 Mg m^−^^3^
Monoclinic, *P*2_1_/*n*	Mo *K*α radiation, λ = 0.71073 Å
Hall symbol: -P 2yn	Cell parameters from 14322 reflections
*a* = 7.6830 (4) Å	θ = 3.5–28.0°
*b* = 9.1597 (4) Å	µ = 0.24 mm^−^^1^
*c* = 26.1083 (12) Å	*T* = 293 K
β = 91.683 (4)°	Plate, colourless
*V* = 1836.55 (15) Å^3^	0.3 × 0.2 × 0.2 mm
*Z* = 4	

### Data collection

**Table:**

Oxford Diffraction Xcalibur Sapphire3 diffractometer	3616 independent reflections
Radiation source: fine-focus sealed tube	2922 reflections with *I* > 2σ(*I*)
Graphite monochromator	*R*_int_ = 0.037
Detector resolution: 16.1049 pixels mm^-1^	θ_max_ = 26.0°, θ_min_ = 3.5°
ω scan	*h* = −9→9
Absorption correction: multi-scan (*CrysAlis PRO*; Oxford Diffraction, 2010)	*k* = −11→11
*T*_min_ = 0.832, *T*_max_ = 1.000	*l* = −32→32
27359 measured reflections	

### Refinement

**Table:**

Refinement on *F*^2^	Primary atom site location: structure-invariant direct methods
Least-squares matrix: full	Secondary atom site location: difference Fourier map
*R*[*F*^2^ > 2σ(*F*^2^)] = 0.048	Hydrogen site location: inferred from neighbouring sites
*wR*(*F*^2^) = 0.112	H atoms treated by a mixture of independent and constrained refinement
*S* = 1.12	*w* = 1/[σ^2^(*F*_o_^2^) + (0.0328*P*)^2^ + 1.1459*P*] where *P* = (*F*_o_^2^ + 2*F*_c_^2^)/3
3616 reflections	(Δ/σ)_max_ < 0.001
256 parameters	Δρ_max_ = 0.21 e Å^−^^3^
0 restraints	Δρ_min_ = −0.23 e Å^−^^3^

### Special details

**Table:**

Experimental. *CrysAlis PRO*, Oxford Diffraction Ltd., Version 1.171.34.40 (release 27–08-2010 CrysAlis171. NET) (compiled Aug 27 2010,11:50:40) Empirical absorption correction using spherical harmonics, implemented in SCALE3 ABSPACK scaling algorithm.
Geometry. All e.s.d.'s (except the e.s.d. in the dihedral angle between two l.s. planes) are estimated using the full covariance matrix. The cell e.s.d.'s are taken into account individually in the estimation of e.s.d.'s in distances, angles and torsion angles; correlations between e.s.d.'s in cell parameters are only used when they are defined by crystal symmetry. An approximate (isotropic) treatment of cell e.s.d.'s is used for estimating e.s.d.'s involving l.s. planes.
Refinement. Refinement of *F*^2^ against ALL reflections. The weighted *R*-factor *wR* and goodness of fit *S* are based on *F*^2^, conventional *R*-factors *R* are based on *F*, with *F* set to zero for negative *F*^2^. The threshold expression of *F*^2^ > σ(*F*^2^) is used only for calculating *R*-factors(gt) *etc*. and is not relevant to the choice of reflections for refinement. *R*-factors based on *F*^2^ are statistically about twice as large as those based on *F*, and *R*- factors based on ALL data will be even larger.

### Fractional atomic coordinates and isotropic or equivalent isotropic displacement parameters (Å^2^)

**Table:**

	*x*	*y*	*z*	*U*_iso_*/*U*_eq_	
Cl1	0.85943 (10)	0.62565 (8)	0.12909 (3)	0.0738 (2)	
C1	0.2793 (3)	−0.0901 (2)	−0.06670 (7)	0.0343 (4)	
O1	0.43612 (17)	−0.10159 (17)	−0.05818 (5)	0.0413 (4)	
N2	0.2112 (2)	−0.0929 (2)	−0.11646 (6)	0.0417 (4)	
O3	0.03330 (18)	−0.13060 (17)	−0.12029 (6)	0.0459 (4)	
C4	−0.0602 (3)	−0.0201 (4)	−0.14807 (11)	0.0706 (8)	
H4B	−0.1812	−0.0459	−0.1506	0.106*	
H4A	−0.0148	−0.0116	−0.1818	0.106*	
H4C	−0.0474	0.0714	−0.1305	0.106*	
N5	0.1626 (2)	−0.0688 (2)	−0.03072 (6)	0.0394 (4)	
N6	0.2138 (2)	−0.0411 (2)	0.02087 (7)	0.0408 (4)	
C7	0.3083 (3)	−0.1280 (2)	−0.16026 (8)	0.0404 (5)	
C8	0.2614 (3)	−0.2474 (3)	−0.19022 (9)	0.0556 (6)	
H8	0.1703	−0.3074	−0.1806	0.067*	
C9	0.3496 (4)	−0.2774 (3)	−0.23418 (10)	0.0668 (8)	
H9	0.3177	−0.3571	−0.2544	0.080*	
C10	0.4843 (4)	−0.1895 (4)	−0.24800 (9)	0.0648 (8)	
H10	0.5456	−0.2110	−0.2772	0.078*	
C11	0.5297 (3)	−0.0690 (3)	−0.21873 (8)	0.0534 (6)	
H11	0.6205	−0.0094	−0.2289	0.064*	
C12	0.4424 (3)	−0.0350 (3)	−0.17444 (7)	0.0409 (5)	
C13	0.4906 (3)	0.0949 (3)	−0.14239 (8)	0.0453 (5)	
H131	0.3875	0.1335	−0.1266	0.054*	
H132	0.5729	0.0662	−0.1154	0.054*	
O13	0.5663 (2)	0.2046 (2)	−0.17350 (6)	0.0566 (4)	
C14	0.6187 (3)	0.3245 (3)	−0.14708 (9)	0.0480 (6)	
N15	0.6511 (2)	0.3234 (2)	−0.09724 (7)	0.0454 (4)	
N16	0.7001 (2)	0.4639 (2)	−0.08603 (7)	0.0464 (5)	
C17	0.6965 (4)	0.5466 (3)	−0.12880 (11)	0.0653 (7)	
H17	0.7243	0.6453	−0.1305	0.078*	
C18	0.6457 (4)	0.4619 (3)	−0.16864 (11)	0.0636 (7)	
H18	0.6318	0.4889	−0.2029	0.076*	
C19	0.7379 (3)	0.5041 (2)	−0.03455 (9)	0.0425 (5)	
C20	0.6784 (3)	0.4182 (2)	0.00479 (9)	0.0425 (5)	
H20	0.6131	0.3349	−0.0027	0.051*	
C21	0.7157 (3)	0.4559 (2)	0.05512 (9)	0.0464 (5)	
H21	0.6777	0.3975	0.0817	0.056*	
C22	0.8103 (3)	0.5816 (3)	0.06560 (10)	0.0504 (6)	
C23	0.8675 (3)	0.6674 (3)	0.02681 (11)	0.0584 (7)	
H23	0.9305	0.7517	0.0344	0.070*	
C24	0.8325 (3)	0.6298 (3)	−0.02355 (11)	0.0548 (6)	
H24	0.8720	0.6882	−0.0499	0.066*	
H61	0.305 (3)	0.013 (3)	0.0208 (8)	0.045 (7)*	
H62	0.251 (3)	−0.123 (3)	0.0344 (10)	0.053 (7)*	
H51	0.053 (3)	−0.051 (3)	−0.0385 (9)	0.051 (7)*	

### Atomic displacement parameters (Å^2^)

**Table:**

	*U*^11^	*U*^22^	*U*^33^	*U*^12^	*U*^13^	*U*^23^
Cl1	0.0739 (5)	0.0668 (5)	0.0798 (5)	−0.0013 (4)	−0.0113 (4)	−0.0195 (4)
C1	0.0345 (11)	0.0338 (10)	0.0345 (10)	−0.0024 (8)	0.0004 (8)	0.0022 (8)
O1	0.0298 (7)	0.0558 (9)	0.0381 (8)	0.0005 (7)	−0.0013 (6)	0.0025 (7)
N2	0.0273 (8)	0.0621 (12)	0.0357 (9)	−0.0054 (8)	−0.0017 (7)	−0.0022 (8)
O3	0.0298 (7)	0.0595 (10)	0.0481 (9)	−0.0076 (7)	−0.0048 (6)	0.0012 (7)
C4	0.0473 (15)	0.100 (2)	0.0640 (17)	0.0128 (15)	−0.0123 (12)	0.0124 (16)
N5	0.0324 (9)	0.0516 (11)	0.0342 (9)	0.0018 (8)	−0.0015 (7)	−0.0015 (8)
N6	0.0385 (10)	0.0487 (12)	0.0350 (10)	−0.0009 (10)	−0.0010 (8)	−0.0004 (9)
C7	0.0348 (11)	0.0544 (13)	0.0315 (10)	0.0046 (10)	−0.0055 (8)	−0.0015 (9)
C8	0.0558 (15)	0.0615 (16)	0.0491 (14)	−0.0044 (12)	−0.0036 (11)	−0.0096 (12)
C9	0.0723 (18)	0.0781 (19)	0.0495 (15)	0.0057 (16)	−0.0056 (13)	−0.0237 (14)
C10	0.0602 (16)	0.097 (2)	0.0370 (13)	0.0140 (16)	0.0012 (11)	−0.0177 (14)
C11	0.0413 (12)	0.0837 (19)	0.0352 (11)	0.0048 (12)	−0.0002 (9)	−0.0012 (12)
C12	0.0325 (10)	0.0583 (13)	0.0315 (10)	0.0065 (10)	−0.0032 (8)	−0.0008 (10)
C13	0.0417 (12)	0.0565 (14)	0.0379 (11)	−0.0034 (10)	0.0058 (9)	0.0032 (10)
O13	0.0651 (11)	0.0650 (11)	0.0401 (9)	−0.0129 (9)	0.0058 (8)	0.0076 (8)
C14	0.0415 (12)	0.0543 (14)	0.0486 (13)	0.0016 (10)	0.0084 (10)	0.0117 (11)
N15	0.0462 (11)	0.0394 (10)	0.0509 (11)	−0.0045 (8)	0.0033 (8)	0.0064 (8)
N16	0.0448 (11)	0.0346 (10)	0.0603 (12)	0.0004 (8)	0.0084 (9)	0.0097 (9)
C17	0.0774 (19)	0.0471 (15)	0.0722 (18)	−0.0012 (13)	0.0139 (15)	0.0197 (14)
C18	0.0702 (17)	0.0653 (17)	0.0558 (16)	0.0017 (14)	0.0075 (13)	0.0215 (14)
C19	0.0344 (11)	0.0321 (11)	0.0612 (14)	0.0038 (9)	0.0066 (10)	0.0039 (10)
C20	0.0354 (11)	0.0301 (10)	0.0623 (14)	0.0002 (9)	0.0062 (10)	−0.0001 (10)
C21	0.0397 (12)	0.0377 (12)	0.0622 (15)	0.0043 (10)	0.0054 (10)	0.0019 (11)
C22	0.0400 (12)	0.0404 (12)	0.0708 (16)	0.0064 (10)	0.0005 (11)	−0.0076 (12)
C23	0.0481 (14)	0.0370 (13)	0.090 (2)	−0.0070 (11)	0.0034 (13)	−0.0050 (13)
C24	0.0499 (14)	0.0357 (12)	0.0793 (18)	−0.0051 (11)	0.0109 (12)	0.0061 (12)

### Geometric parameters (Å, º)

**Table:**

Cl1—C22	1.737 (3)	C12—C13	1.495 (3)
C1—O1	1.223 (2)	C13—O13	1.427 (3)
C1—N5	1.332 (3)	C13—H131	0.9700
C1—N2	1.387 (2)	C13—H132	0.9700
N2—O3	1.411 (2)	O13—C14	1.352 (3)
N2—C7	1.420 (3)	C14—N15	1.318 (3)
O3—C4	1.427 (3)	C14—C18	1.397 (3)
C4—H4B	0.9600	N15—N16	1.370 (3)
C4—H4A	0.9600	N16—C17	1.349 (3)
C4—H4C	0.9600	N16—C19	1.415 (3)
N5—N6	1.415 (2)	C17—C18	1.346 (4)
N5—H51	0.88 (2)	C17—H17	0.9300
N6—H61	0.86 (2)	C18—H18	0.9300
N6—H62	0.87 (3)	C19—C20	1.382 (3)
C7—C8	1.386 (3)	C19—C24	1.387 (3)
C7—C12	1.395 (3)	C20—C21	1.380 (3)
C8—C9	1.378 (4)	C20—H20	0.9300
C8—H8	0.9300	C21—C22	1.385 (3)
C9—C10	1.368 (4)	C21—H21	0.9300
C9—H9	0.9300	C22—C23	1.364 (4)
C10—C11	1.382 (4)	C23—C24	1.378 (4)
C10—H10	0.9300	C23—H23	0.9300
C11—C12	1.389 (3)	C24—H24	0.9300
C11—H11	0.9300		
			
O1—C1—N5	124.34 (18)	O13—C13—H131	109.7
O1—C1—N2	120.78 (18)	C12—C13—H131	109.7
N5—C1—N2	114.83 (17)	O13—C13—H132	109.7
C1—N2—O3	114.16 (16)	C12—C13—H132	109.7
C1—N2—C7	124.46 (16)	H131—C13—H132	108.2
O3—N2—C7	114.70 (15)	C14—O13—C13	113.70 (17)
N2—O3—C4	109.54 (17)	N15—C14—O13	122.9 (2)
O3—C4—H4B	109.5	N15—C14—C18	112.3 (2)
O3—C4—H4A	109.5	O13—C14—C18	124.9 (2)
H4B—C4—H4A	109.5	C14—N15—N16	104.28 (18)
O3—C4—H4C	109.5	C17—N16—N15	110.6 (2)
H4B—C4—H4C	109.5	C17—N16—C19	129.7 (2)
H4A—C4—H4C	109.5	N15—N16—C19	119.64 (17)
C1—N5—N6	121.57 (17)	C18—C17—N16	108.3 (2)
C1—N5—H51	121.9 (15)	C18—C17—H17	125.8
N6—N5—H51	115.1 (15)	N16—C17—H17	125.8
N5—N6—H61	107.8 (15)	C17—C18—C14	104.5 (2)
N5—N6—H62	108.1 (16)	C17—C18—H18	127.7
H61—N6—H62	104 (2)	C14—C18—H18	127.7
C8—C7—C12	120.9 (2)	C20—C19—C24	120.1 (2)
C8—C7—N2	120.0 (2)	C20—C19—N16	119.6 (2)
C12—C7—N2	119.00 (19)	C24—C19—N16	120.3 (2)
C9—C8—C7	120.1 (2)	C21—C20—C19	120.1 (2)
C9—C8—H8	120.0	C21—C20—H20	120.0
C7—C8—H8	120.0	C19—C20—H20	120.0
C10—C9—C8	119.8 (3)	C20—C21—C22	119.3 (2)
C10—C9—H9	120.1	C20—C21—H21	120.4
C8—C9—H9	120.1	C22—C21—H21	120.4
C9—C10—C11	120.4 (2)	C23—C22—C21	120.7 (2)
C9—C10—H10	119.8	C23—C22—Cl1	120.6 (2)
C11—C10—H10	119.8	C21—C22—Cl1	118.7 (2)
C10—C11—C12	121.2 (2)	C22—C23—C24	120.4 (2)
C10—C11—H11	119.4	C22—C23—H23	119.8
C12—C11—H11	119.4	C24—C23—H23	119.8
C11—C12—C7	117.6 (2)	C23—C24—C19	119.4 (2)
C11—C12—C13	121.8 (2)	C23—C24—H24	120.3
C7—C12—C13	120.64 (18)	C19—C24—H24	120.3
O13—C13—C12	109.90 (17)		
			
O1—C1—N2—O3	158.54 (18)	C13—O13—C14—N15	21.2 (3)
N5—C1—N2—O3	−23.9 (3)	C13—O13—C14—C18	−159.2 (2)
O1—C1—N2—C7	8.8 (3)	O13—C14—N15—N16	179.8 (2)
N5—C1—N2—C7	−173.6 (2)	C18—C14—N15—N16	0.1 (3)
C1—N2—O3—C4	125.3 (2)	C14—N15—N16—C17	0.0 (2)
C7—N2—O3—C4	−81.9 (2)	C14—N15—N16—C19	177.28 (18)
O1—C1—N5—N6	7.2 (3)	N15—N16—C17—C18	−0.2 (3)
N2—C1—N5—N6	−170.22 (19)	C19—N16—C17—C18	−177.1 (2)
C1—N2—C7—C8	118.9 (2)	N16—C17—C18—C14	0.3 (3)
O3—N2—C7—C8	−30.7 (3)	N15—C14—C18—C17	−0.2 (3)
C1—N2—C7—C12	−65.6 (3)	O13—C14—C18—C17	−179.9 (2)
O3—N2—C7—C12	144.89 (18)	C17—N16—C19—C20	157.9 (2)
C12—C7—C8—C9	1.2 (4)	N15—N16—C19—C20	−18.7 (3)
N2—C7—C8—C9	176.6 (2)	C17—N16—C19—C24	−21.4 (3)
C7—C8—C9—C10	0.5 (4)	N15—N16—C19—C24	161.9 (2)
C8—C9—C10—C11	−1.5 (4)	C24—C19—C20—C21	−1.2 (3)
C9—C10—C11—C12	0.9 (4)	N16—C19—C20—C21	179.50 (19)
C10—C11—C12—C7	0.6 (3)	C19—C20—C21—C22	1.2 (3)
C10—C11—C12—C13	179.9 (2)	C20—C21—C22—C23	−0.4 (3)
C8—C7—C12—C11	−1.7 (3)	C20—C21—C22—Cl1	−178.87 (16)
N2—C7—C12—C11	−177.21 (19)	C21—C22—C23—C24	−0.3 (4)
C8—C7—C12—C13	179.1 (2)	Cl1—C22—C23—C24	178.14 (19)
N2—C7—C12—C13	3.6 (3)	C22—C23—C24—C19	0.2 (4)
C11—C12—C13—O13	27.3 (3)	C20—C19—C24—C23	0.5 (3)
C7—C12—C13—O13	−153.51 (19)	N16—C19—C24—C23	179.8 (2)
C12—C13—O13—C14	−178.05 (18)		

### Hydrogen-bond geometry (Å, º)

**Table:**

*D*—H···*A*	*D*—H	H···*A*	*D*···*A*	*D*—H···*A*
N5—H51···N6^i^	0.88 (2)	2.28 (2)	3.080 (3)	153 (2)
N6—H61···O1^ii^	0.86 (2)	2.34 (2)	3.120 (3)	152 (2)
N6—H62···N15^ii^	0.87 (3)	2.56 (3)	3.408 (3)	164 (2)

Symmetry codes: (i) −*x*, −*y*, −*z*; (ii) −*x*+1, −*y*, −*z*.
